# Digital image analysis using video microscopy of human-derived prostate cancer vs normal prostate organoids to assess migratory behavior on extracellular matrix proteins

**DOI:** 10.3389/fonc.2022.1083150

**Published:** 2023-01-13

**Authors:** Kendra D. Marr, Natalia A. Ignatenko, Noel A. Warfel, Ken Batai, Anne E. Cress, Grant R. Pollock, Ava C. Wong, Benjamin R. Lee

**Affiliations:** ^1^ Cancer Biology, University of Arizona, Tucson, AZ, United States; ^2^ MD/PhD Program, College of Medicine Tucson, University of Arizona, Tucson, AZ, United States; ^3^ Cellular & Molecular Medicine, University of Arizona, Tucson, AZ, United States; ^4^ Cancer Prevention & Control, Roswell Park Comprehensive Cancer Center, Buffalo, NY, United States; ^5^ Urology, College of Medicine Tucson, University of Arizona, Tucson, AZ, United States; ^6^ College of Nursing, University of Arizona, Tucson, AZ, United States

**Keywords:** organoids, prostate cancer, video microscopy, cancer phenotypes, extracellular matrix

## Abstract

The advent of perpetuating living organoids derived from patient tissue is a promising avenue for cancer research but is limited by difficulties with precise characterization. In this brief communication, we demonstrate *via* time-lapse imaging distinct phenotypes of prostate organoids derived from patient material– without confirmation of cellular identity. We show that organoids derived from histologically normal tissue more readily spread on a physiologic extracellular matrix (ECM) than on pathologic ECM (p<0.0001), while tumor-derived organoids spread equally on either substrate (p=0.2406). This study is an important proof-of-concept to defer precise characterization of organoids and still glean information into disease pathology.

## 1 Introduction

Patient-derived organoids (PDOs) provide a method for investigating human prostate cancer cells in culture in three dimensions. There remains a paucity of biobanked low to intermediate-grade PDOs. This is due, in part, to difficulty in confirming cell(s)-of-origin, and this cancer’s slow-growing nature leads to low yield sample volumes when grown from needle biopsies (1). The goal of this study was to define distinct phenotypes of prostate PDOs grown from tumor or normal samples without regard to the identity of the cells within the final organoids. Our approach was to investigate the phenotypic behavior of living organoids, obtained using needle biopsies from gross normal and prostate cancer tissue, when exposed to physiologically relevant extracellular matrix (ECM) proteins. Prostate tumors undergo ECM composition change, budding through areas of basal cell-secreted Laminin-332 loss to migrate along nerves and stroma that are rich in Laminin-511 (2, 3). Through this route, the cancer escapes the gland and accesses the lymph nodes, pelvic skeleton, and vertebral spine (4, 5). We hypothesize that these early cell-ECM interactions can provide prognostic indications of prostate tumor behavior before it escapes its primary site. Here, we utilize digital image analysis *via* a 3D video imaging system to directly observe and record morphologic transformations of the organoids within 21 days post-explant.

## 2 Methods

### 2.1 Patient cohort

Fresh biospecimens from men treated at Banner University Medical Center (Tucson, AZ) were procured under IRB approval. The cohort included only hormone-naïve prostate cancer patients with localized disease who underwent robotic radical prostatectomy (RP) as part of the standard of care. Informed consent was obtained for all patients. Samples and redacted pathology reports were biobanked in the University of Arizona Tissue Acquisition and Cellular/Molecular Analysis Shared Resource. All were stripped of personal health information and identifiers.

### 2.2 Organoids

Needle cores from fresh RP specimens were harvested at the time of surgery. Both the primary tumor and non-diseased regions of the prostate were sampled based on gross morphology so that each tumor sample has a matched normal control. Repeat needle cores histologically confirmed gross morphology. PDOs were processed as described in (6). 3D culture of RWPE1 spheroids was performed as described in (7).

### 2.3 Live imaging

Glass-bottom dishes (MatTek P35G-1.5-14-C) were coated with Laminin-332 or Laminin-511 as described in (8). Organoids were isolated from Matrigel by pipetting up and down with a wide-bore tip and centrifuging 400xG for 5min, then plated on the pre-coated dishes. Time-lapse images were acquired every hour for up to 24hr on a Nikon Eclipse Ti2-E in a regulated environmental chamber set to 37°C, 21% O_2_, 5% CO_2_, then processed into binary masks for analysis using ImageJ and GraphPad Prism 8. Spreading is estimated by C = 4*pi*A/P^2, where C is circularity, A is area and P is perimeter. We defined “spread” as C < 0.60.

## 3 Results

**Figure 1:**
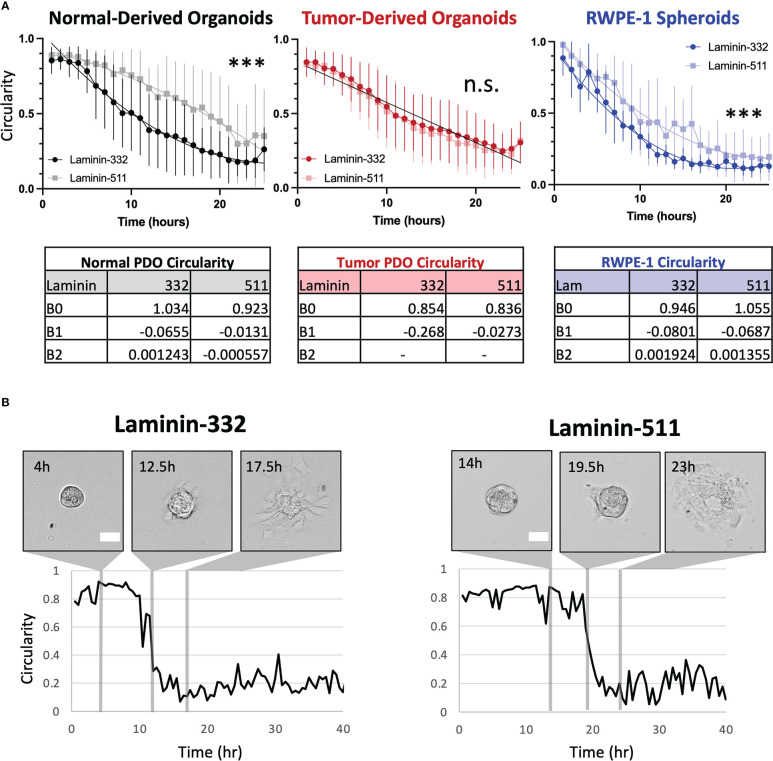
**(A)** Organoids from normal tissue spread more quickly on Laminin-332 than on Laminin-511 substrate. Line plots of average circularity +/- SD (error bars) per substrate for normal- (grayscale) and tumor-derived (red) organoids from 3 patients and RWPE-1 (blue) spheroids. Nonlinear fitted curves are plotted on the normal graph (dark line, Lam-332; light line, Lam-511) ***p<0.0001, n.s = not significant. For tumor-derived organoids, substrate-specific circularity curves are not significantly different (p=0.2406), and therefore a single, global curve is plotted (black line). Time to spreading was interpolated from the resultant curves using C = 0.60. Normal Lam-332, n = 24; Normal Lam-511, n = 7; Tumor Lam-332, n = 29; Tumor Lam-511, n = 23; RWPE-1 Lam-332 n= 6, RWPE-1 Lam511 n = 7. No outliers were detected in PDO samples, one detected and removed from RWPE-1 spheroid measurements (ROUT Q=1%). **(B)** Representative images with corresponding circularity measurements plotted over time for organoids derived from the normal prostate regions of one patient on either Laminin-332 or Laminin-511. Scale bar = 100μm.

Our institution derived organoids from needle core samples of primary tumors and matched normal tissue from 56 prostate cancer patients. For the current study we chose organoids from three patients whose tumors exhibited similar pathology (Grade Group II, pT3aN0). We measured PDO spreading onto two ECM proteins: Laminin-332, normally secreted by prostate basal epithelial cells, and Laminin-511, which coats smooth muscle cells and nerve axons within the gland (2). Average circularity (C) over time was fitted to a nonlinear curve as a proxy for spreading. Circularity curves are significantly different for normal-derived organoids depending on their substrate, with an average time to spreading of 8.37hr on Laminin-332 and 14.29hr on Laminin-511 when interpolated using C=0.60 (p<0.0001). Interestingly, curves of tumor-derived organoids do not differ between substrates, time to spreading is 9.49hr on Laminin-332 and 8.64hr on Laminin-511 (p=0.2406) (**Figure 1**; **Figure S1**). We compared the above results to laboratory-generated prostate spheroids grown from RWPE1, a nontumorigenic, immortalized line derived from epithelial cells of a histologically normal adult human prostate (9). The behavior of RWPE-1 spheroids mirrored that of the normal-derived PDOs by spreading more quickly on Laminin-332 (4.49hr) compared to Laminin-511 (8.91hr, p<0.0001).

## 4 Discussion

We show that the transition from the mature glandular structure of the organoid to a state of heightened cell motility and escape is influenced by the composition of the available ECM, whereas normal-derived organoids spread more quickly on Laminin-332 (**Supplementary Movie 1**), the isoform encountered in intact prostate glands, than on the stromal isoform Laminin-511 (**Supplementary Movie 2**). In contrast, tumor-derived organoids display no ECM preference, likely reflecting their adaptation to the stromal microenvironment. It will be interesting to correlate invasive behavior to the clinicopathologic grading and staging of the primary neoplasm. We predict that organoids derived from higher grade tumors will spread more readily on Laminin-511, reflective of their propensity to invade through the smooth muscle stroma along nerves to escape the prostate gland (4, 5). We further predict this to correlate to metastatic potential, as local invasion precedes extra-prostatic extension.

Characterization of organoids is an ongoing barrier to performing rapid and cost-effective studies utilizing this powerful biological system. This study demonstrates the intriguing possibility of gleaning information on tumor behavior without the need for costly analyses to confirm cell-of-origin. We assert that measurable phenotypic differences are perceptible whether the origin of tumor-derived organoids is truly a cancer cell or neighboring healthy cell. We believe that this approach with be a boon towards understanding early aggressive events that dictate disease outcomes.

## Data availability statement

The raw data supporting the conclusions of this article will be made available by the authors, without undue reservation.

## Ethics statement

The studies involving human participants were reviewed and approved by Institutional Review Board - The University of Arizona Human Subjects Protection Program. The patients/participants provided their written informed consent to participate in this study.

## Author contributions

KM, NI, KB, AC, and BL jointly conceptualized the project. NI, GP, and BL generated the organoid material used in this project. KM performed experiments and generated the data used in this manuscript with technical assistance from NI and NW, KM drafted this manuscript, with edits and review provided by all co-authors. All authors contributed to the article and approved the submitted version.
