# Supplementary figures and images for: Digital image analysis using video microscopy of human-derived prostate cancer vs normal prostate organoids to assess migratory behavior on extracellular matrix proteins
Source: Front Oncol. 2023 Jan 13;12:1083150. doi: 10.3389/fonc.2022.1083150 (PMC9885251; doi:10.3389/fonc.2022.1083150)

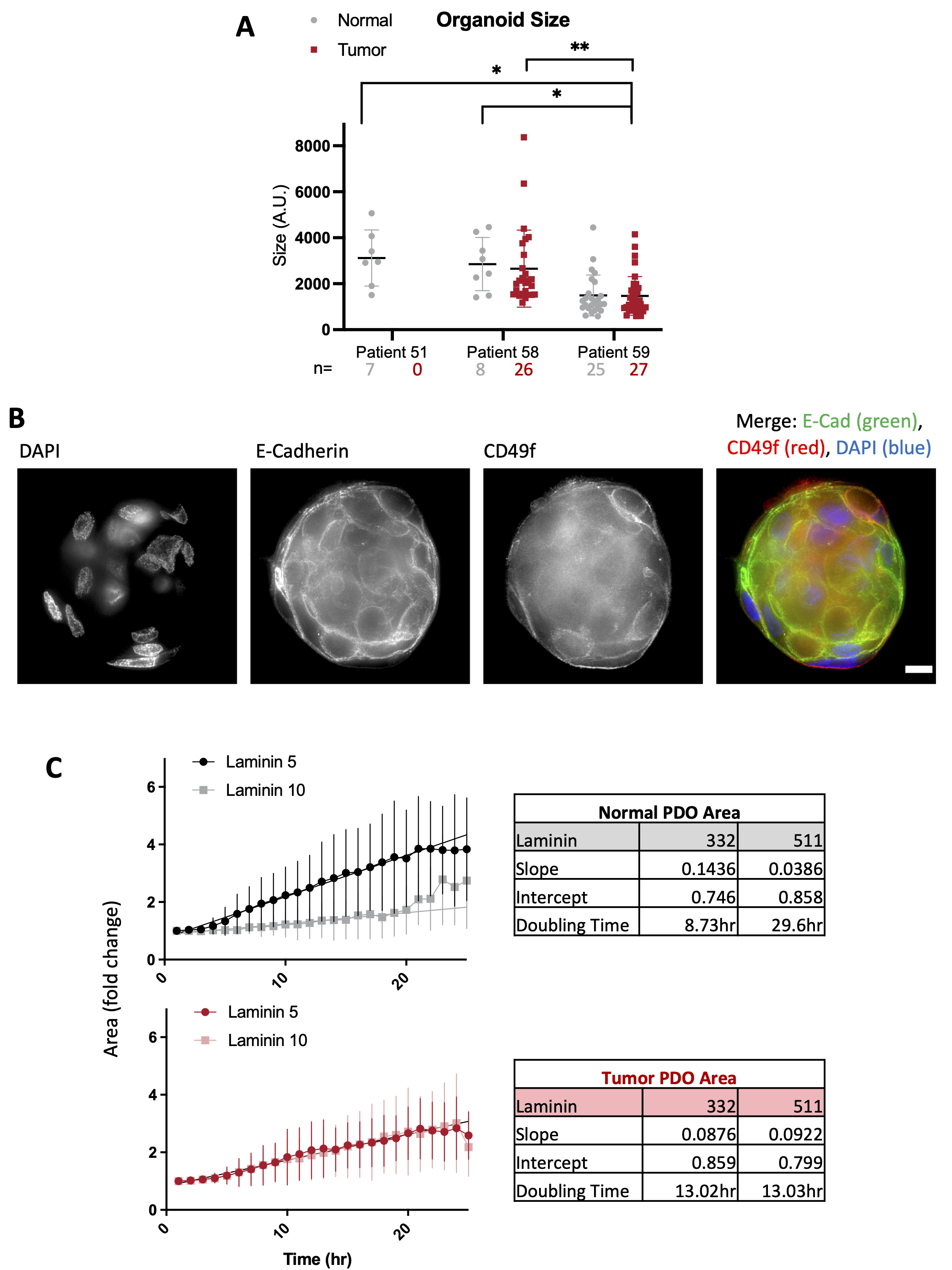

Supplement: Supplementary file 1 [file Image_1.tiff]
