# Supplementary material for: Digital image analysis using video microscopy of human-derived prostate cancer vs normal prostate organoids to assess migratory behavior on extracellular matrix proteins
Source: Front Oncol. 2023 Jan 13;12:1083150. doi: 10.3389/fonc.2022.1083150 (PMC9885251; doi:10.3389/fonc.2022.1083150)
Supplement: Supplementary file 2 [file Table_1.docx]

**Supplemental information:**

Organoid size at the beginning of the experiment varied by patient but not by tissue type. Both tumor-derived (1 472 sq.px.; p<0.01) and normal-derived (1 491 sq.px.; p<0.05) organoids from Patient 59 were smaller on average than those from Patient 51 (normal-derived 3 117 sq.px.) or Patient 58 (normal-derived 2 854 sq.px., tumor-derived 2 642 sq.px.). The decrease in circularity plotted in these PDOs is inversely related to surface area of the binary masks in the processed videos, reflecting the spreading from compact 3D organoids to a flattened sheet of cells onto a 2D surface. It took 8.73hr for normal-derived organoids to double in surface area on Laminin-332 versus 29.6hr on Laminin-511. For tumor-derived organoids, these times are 13.02hr and 13.03hr, respectively. Each organoid demonstrated cell-cell distribution of epithelial E-Cadherin and the prostate basal cell marker CD49f.

**Supplemental Methods**

**3D indirect immunofluorescence microscopy**

Organoids were isolated as above and fixed in 4% paraformaldehyde, 1% Triton X-100 in PBS overnight at 4°C. They were incubated in 50mM NH_4_Cl in PBS for 30min, permeabilized with 0.1% Triton X-100 in PBS for 30min, and then blocked with IF buffer (3% BSA, 0.1% Triton X-100 in PBS) for 1hr at room temperature. Primary antibodies (anti-E-Cad M168 1:100, anti-CD49f J1B5 1:100) in IF buffer were incubated for 48hr at 4°C. Alexa-fluor conjugated secondary antibodies (1:1 000) were applied overnight at 4°C. Samples were mounted with ProLong Diamond with DAPI (Thermofisher P36962).

**Supplemental Figure 1:** (A) Mean organoid size differs by patient, but not interpatient normal-derived versus tumor-derived. Scatter plot of individual organoid sizes measured as area in pixels with mean (black lines) and standard deviation (error bars). Patient 51 n=7 normal, n=0 tumor; Patient 58 n=8 normal, 26 tumor; Patient 59 n=25 normal, n=27 tumor. *p<0.05; **p<0.01, two-tailed t-test with unequal variance. Tumor-derived organoids from Patient 51 did not survive the analysis and were excluded. (B) Organoids have molecular markers of prostate glands. Indirect immunofluorescence microscopy against DAPI, E-Cadherin and CD49f. Merged image (right) illustrates distribution of DAPI (blue), E-Cad (green), and CD49f (red), consistent with an epithelial layer containing peripheral basal cells. Organoid is from Patient 59 tumor. Scale bar = 10µm.

**Supplemental Video 1:** Representative video of a normal-derived organoid on Laminin-332.

**Supplemental Video 2:** Representative video of a normal-derived organoid on Laminin-511.

**Supplemental Video 3:** Representative video of a tumor-derived organoid on Laminin-332.

**Supplemental Video 4:** Representative video of a tumor-derived organoid on Laminin-511.

**Supplemental Video 5:** Representative video of an RWPE-1 spheroid on Laminin-332.

**Supplemental Video 6:** Representative video of an RWPE-1 spheroid on Laminin-511.
